# HMGA2 Contributes to Distant Metastasis and Poor Prognosis by Promoting Angiogenesis in Oral Squamous Cell Carcinoma

**DOI:** 10.3390/ijms20102473

**Published:** 2019-05-19

**Authors:** Junki Sakata, Akiyuki Hirosue, Ryoji Yoshida, Kenta Kawahara, Yuichiro Matsuoka, Tatsuro Yamamoto, Masafumi Nakamoto, Masatoshi Hirayama, Nozomu Takahashi, Takuya Nakamura, Hidetaka Arita, Hikaru Nakashima, Masashi Nagata, Akimitsu Hiraki, Masanori Shinohara, Hideki Nakayama

**Affiliations:** 1 Department of Oral and Maxillofacial Surgery, Faculty of Life Sciences, Kumamoto University, Kumamoto 860-8556, Japan; j.sakata0510@gmail.com (J.S.); ryoshida@kumamoto-u.ac.jp (R.Y.); k.k.ronbun@gmail.com (K.K.); kokopelli205@gmail.com (Y.M.); tatsuro.y.1.3@gmail.com (T.Y.); mabo.20jd.jm@gmail.com (M.N.); dd104040@gmail.com (M.H.); n.takahashi6074@gmail.com (N.T.); takuyanakamura2008@gmail.com (T.N.); hidetaka.arita@gmail.com (H.A.); ricken_hikaru@yahoo.co.jp (H.N.); nagatama0213@gmail.com (M.N.); shinora@kumamoto-u.ac.jp (M.S.); hinakaya@kumamoto-u.ac.jp (H.N.); 2 Section of Oral Oncology, Department of Oral and Maxillofacial Surgery, Fukuoka Dental College, Fukuoka 814-0193, Japan; hiluckyhilucky@gmail.com

**Keywords:** angiogenesis, HMGA2, metastasis, oral squamous cell carcinoma, prognosis

## Abstract

The highly malignant phenotype of oral squamous cell carcinoma (OSCC), including the presence of nodal and distant metastasis, reduces patient survival. High-mobility group A protein 2 (HMGA2) is a non-histone chromatin factor that is involved in advanced malignant phenotypes and poor prognosis in several human cancers. However, its biological role in OSCC remains to be elucidated. The purpose of this study was to determine the clinical significance and role of HMGA2 in the malignant potential of OSCC. We first investigated the expression pattern of HMGA2 and its clinical relevance in 110 OSCC specimens using immunohistochemical staining. In addition, we examined the effects HMGA2 on the regulation of vascular endothelial growth factor (VEGF)-A, VEGF-C, and fibroblast growth factor (FGF)-2, which are related to angiogenesis, in vitro. High expression of HMGA2 was significantly correlated with distant metastasis and poor prognosis. Further, HMGA2 depletion in OSCC cells reduced the expression of angiogenesis genes. In OSCC tissues with high HMGA2 expression, angiogenesis genes were increased and a high proportion of blood vessels was observed. These findings suggest that HMGA2 plays a significant role in the regulation of angiogenesis and might be a potential biomarker to predict distant metastasis and prognosis in OSCC.

## 1. Introduction

Oral cancer is one of the most common head and neck neoplasms, and the survival rate of patients with this disease has not improved despite advances in diagnostic techniques and treatments [1,2]. The highly malignant phenotype of oral squamous cell carcinoma (OSCC), including the presence of nodal and distant metastasis at the time of diagnosis, results in poor therapeutic outcomes and reduces patient survival [3]. Therefore, elucidating the crucial molecular pathways associated with the metastasis of OSCC is important to develop more effective treatments and to improve patient survival. 

Cancer metastasis is caused by complex interactions among intrinsic tumor cell properties, as well as interplay between cancer cells and the tumor microenvironment. Moreover, typical associated phenomena include epithelial–mesenchymal transition (EMT) and angiogenesis [4]. EMT is a physiological process that takes place during embryonic development, wound healing, and tumor progression [5,6]. The prominent changes that are characteristic of EMT are the down-regulation of epithelial markers like E-cadherin and the up-regulation of mesenchymal markers like Vimentin [7]. In addition, transcription factors such as Snail, Slug, and Twist, have been confirmed to play important roles in promoting EMT [8,9]. Recent reports have also implicated EMT in the malignant conversion of transformed cells, which is associated with the gain of invasive or metastasizing abilities in multiple types of tumors [10,11,12]. Angiogenesis, defined by the formation of new blood vessels from preexisting ones, is a critical event in tumor progression. Neovascularization is necessary to deliver oxygen and nutrient substances for local tumor growth. Additionally, the increased permeability of the newly formed blood vessels in the tumor microenvironment can facilitate the invasion and metastasis of tumor cells. Angiogenesis has also been demonstrated to closely correlate with tumor stage, metastasis, and poor prognosis in OSCC [13,14,15]. 

High-mobility group A2 (HMGA2), which is a member of the high-mobility group (HMG) family, is a non-histone chromatin protein that participates in many biological processes including cell growth and differentiation [16]. HMGA2 has three AT-hook DNA-binding motifs that mediate binding to the minor groove of AT-rich DNA sequences, and this protein can modulate transcription by changing the chromatin architecture [16]. HMGA2 is highly expressed during embryogenesis and was also found to be overexpressed in a number of tumors [17,18,19,20]. The overexpression of HMGA2 is also correlated with the occurrence of metastasis and poor prognosis in several types of cancers [21]. Furthermore, recent studies have demonstrated that this protein can have an important role in EMT [22,23,24]. However, regarding its association with angiogenesis, only a few studies have shown that HMGA2 is required and sufficient to modulate angiogenesis in endothelial cells and endothelial progenitor cells [25,26]. Although, some reports described the importance of HMGA2 in the poor prognosis of oral cancer [27,28,29,30,31,32], its precise role in OSCC has not been fully elucidated.

The purpose of this study was to determine the clinical significance and role of HMGA2 in the highly malignant potential of OSCC. In the present study, we investigated the expression of HMGA2 in biopsy specimens of OSCC patients through immunohistochemical analysis and found that high HMGA2 expression level was significantly correlated with N-stage, distant metastasis, and poor prognosis. Furthermore, we found that HMGA2 is associated with the transcriptional activation of angiogenesis genes in OSCC cell lines.

## 2. Results

### 2.1. Clinical Significance of HMGA2 Expression in OSCC Tissues 

**Figure 1:**
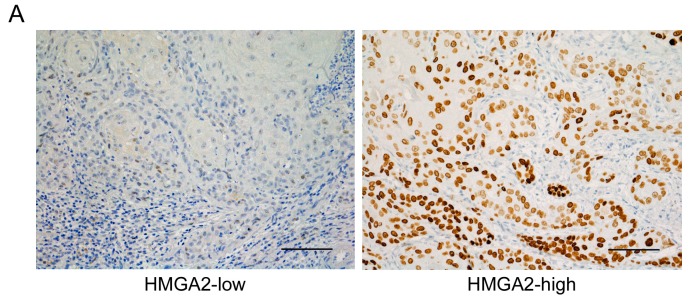

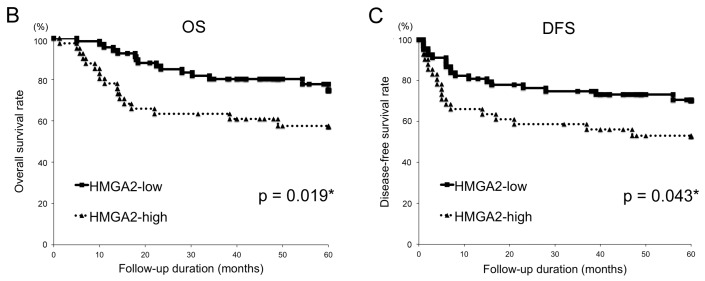
Tumor high-mobility group A protein 2 (HMGA2) expression status affects the survival rate of oral squamous cell carcinoma (OSCC) patients. (**A**) Immunohistochemical staining for HMGA2 in OSCC biopsy specimens. Representative microscopic images are shown according to expression status. Original magnification, ×200, scale bar = 100 μm. (**B**) Based on Kaplan–Meier survival analysis of OSCC, the patients were divided into two groups based on HMGA2 immunostaining scores (low- or high-expression groups). Overall survival (OS) of the 110 OSCC patients based on the status of HMGA2 expression was determined. * *p* < 0.05. (**C**) Disease-free survival (DFS) of the 110 OSCC patients based on the status of HMGA2 expression. * *p* < 0.05.

To examine the role of HMGA2 in OSCC, we investigated expression levels in biopsy specimens obtained from 110 OSCC patients through immunohistochemical staining. HMGA2 immunoreactivity was observed mainly in the nucleus of epithelial cells. The patients were classified into two groups based on the immunostaining score for HMGA2 expression as low or high expression groups (Figure 1A). Results indicated that clinical OSCC had a variety of immunostaining patterns, which are associated with the biological behavior of cancer cells.

To elucidate the clinical significance of HMGA2 expression in OSCC patients, we examined the correlations between protein levels and clinicopathological variables (Table 1). Of the 110 OSCC cases, 42 (38.2%) showed high expression, whereas 68 (61.2%) showed low expression. As shown in Table 1, the expression status of HMGA2 was associated with N-stage (*p* = 0.011) and distant metastasis (*p* < 0.001). In contrast, there were no significant differences in the expression status of HMGA2 according to age, sex, primary site, T-stage, clinical stage, differentiation, and local recurrence. These results suggest that HMGA2 expression is related to lymph node metastasis and distant metastasis.

### 2.2. Relationships between the HMGA2 and Survival Time 

To assess the relationships between the HMGA2 and survival time, overall and disease-free survival of the 110 OSCC patients were analyzed using the Kaplan–Meier method. The overall and disease-free 5-year survival rates of patients with high HMGA2 expression was significantly lower than those for patients with low expression (*p* = 0.019 and 0.044, respectively; Figure 1B,C).

Multivariate analysis using the Cox proportional hazards regression model revealed that HMGA2 expression and N-stage were not significant predictors of OSCC patient survival. In contrast, local recurrence and distant metastasis were significant prognostic factors for this cohort (Table 2).

### 2.3. Role of HMGA2 in the Transcriptional Control of EMT Markers

EMT is involved in tumor metastasis, and it is thought that this process confers invasive features that allow cells to disseminate. HMGA2 can also have an important role in EMT, and recent studies have demonstrated that transforming growth factor β (TGF-β) mediates EMT by inducing HMGA2 [22,23].

To check the expression levels of HMGA2 and EMT markers in OSCC, we performed quantitative RT-PCR (qRT-PCR) analysis. After 48 h of TGF-β1 stimulation, *HMGA2* mRNA was significantly increased in two OSCC cell lines, specifically Ca9-22 and SAS cells. Using the same cDNA samples, *Slug* and *Vimentin* expression were significantly up-regulated. In contrast, we found the significant down-regulation of *E-cadherin* (Supplementary Figure S1). 

**Figure 2:**
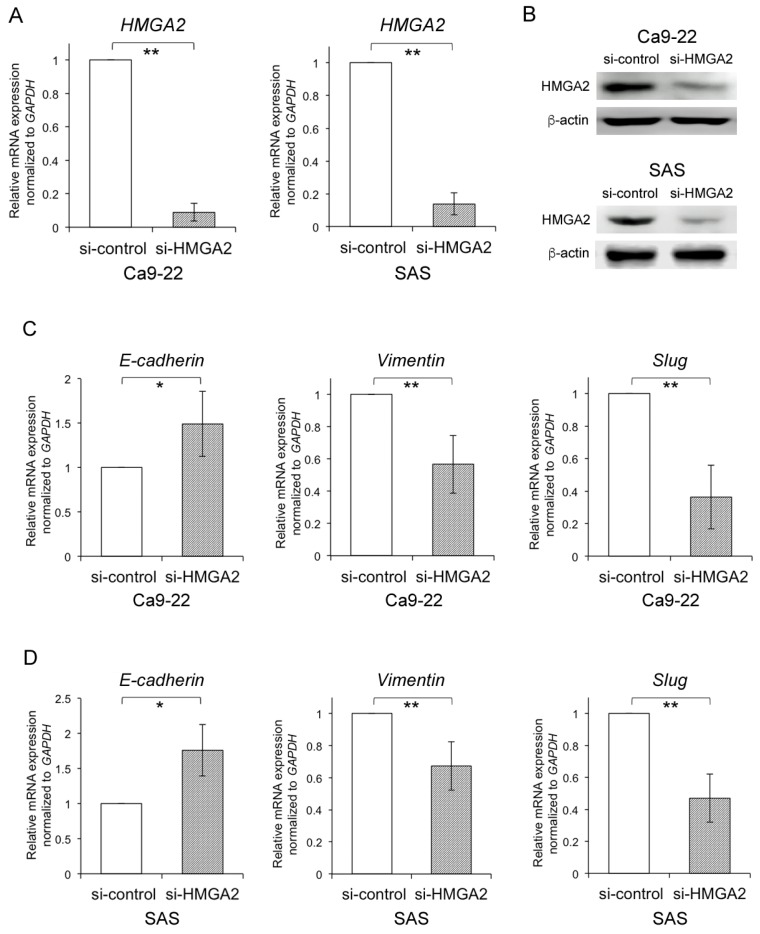
HMGA2 controls the expression of epithelial–mesenchymal transition (EMT) markers in oral squamous cell carcinoma (OSCC) cell lines. (**A**,**B**) The mRNA and protein expression levels of HMGA2 in Ca9-22 cells and SAS cells transfected with HMGA2 siRNA or control. Total RNA and whole-cell lysates were used for qRT-PCR (**A**) and western blot analysis (**B**), respectively. (**C**,**D**) The mRNA expression levels of *E-cadherin*, *Vimentin*, and *Slug* were measured using qRT-PCR with Ca9-22 cells (**C**) and SAS cells (**D**) transfected with si-HMGA2. The values are expressed as the means ± s.d. of triplicate samples. Statistical analysis was performed using a Student’s *t*-test. * *p* < 0.05, ** *p* < 0.01.

In addition, to investigate the transcriptional role of HMGA2 in EMT markers, we performed RNA interference-mediated HMGA2 knockdown using Ca9-22 cells. Western blot and qRT-PCR analyses confirmed that HMGA2 was depleted at both the protein and mRNA levels (Figure 2A,B). Further, *Slug* and *Vimentin* expression were significantly decreased with HMGA2 knockdown. In contrast, we found that *E-cadherin* expression was significantly increased with HMGA2 knockdown (Figure 2C). Similar data were obtained for SAS cells (Figure 2D). 

### 2.4. Involvement of HMGA2 in the EMT Phenotype of OSCC Cells

To determine whether HMGA2 expression affects the phenotype of OSCC cells, we examined morphological changes, proliferation, migration, and invasion in OSCC cells stimulated with TGF-β1. After 48 h of TGF-β1 stimulation, we observed that OSCC cells exhibited a morphological change from the cobblestone form to the spindle shape (Supplementary Figure S2). Cell proliferation was also significantly inhibited by TGF-β1 stimulation but was not changed upon HMGA2 knockdown (Supplementary Figure S3). OSCC cells stimulated with TGF-β1 exhibited obviously enhanced migration toward the wound, compared to that in control cells (Supplementary Figure S4). Moreover, using a Matrigel invasion assay system, we observed enhanced tumor cell invasion in OSCC cells stimulated with TGF-β1 (Supplementary Figure S5). In contrast, the knockdown of HMGA2 did not significantly enhance the migration and invasion activity of OSCC cells (data not shown). These findings suggest that HMGA2 is partially related to the EMT phenotype in OSCC cells. 

### 2.5. Relationship between HMGA2 and Angiogenesis-Associated Genes in OSCC Cells

**Figure 3:**
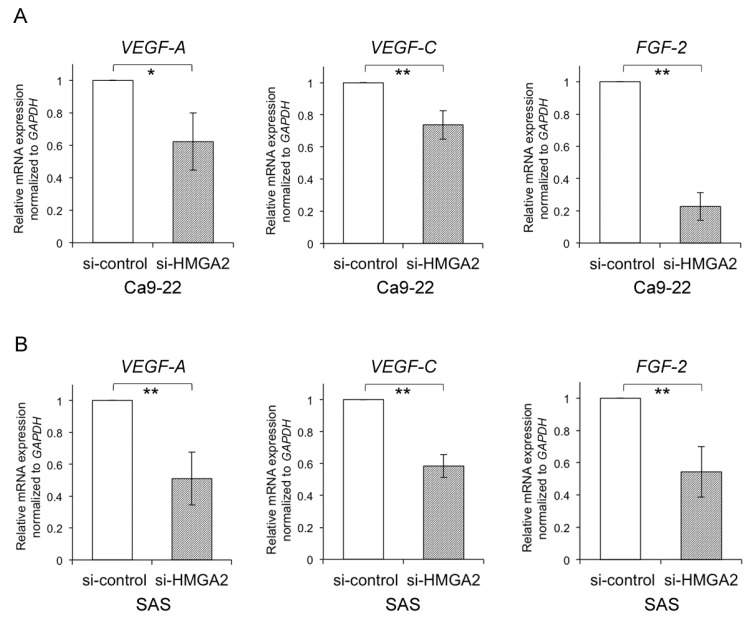
HMGA2 regulates the expression of angiogenesis genes in oral squamous cell carcinoma (OSCC) cell lines. (**A**,**B**) The mRNA expression levels of *VEGF-A*, *VEGF-C*, and *FGF-2* were measured using qRT-PCR in Ca9-22 cells (**A**) and SAS cells (**B**) transfected with si-HMGA2. The values are expressed as the means ± s.d. of triplicate samples. Statistical analysis was performed using the Student’s *t*-test. * *p* < 0.05, ** *p* < 0.01.

To investigate the contribution of HMGA2 to metastasis, we focused on angiogenesis, which plays an essential role in the metastasis of various cancers [14]. However, it remains unclear whether HMGA2 regulates angiogenesis-associated genes in cancer cells. To determine the mRNA expression levels of *VEGF-A*, *VEGF-C*, and *FGF-2*, as angiogenesis-associated genes, upon HMGA2 knockdown, quantitative RT-PCR was performed using the OSCC cells. The expression of *VEGF-A*, *VEGF-C*, and *FGF-2* was significantly up-regulated with siRNA-mediated HMGA2 knockdown in Ca9-22 cells (Figure 3A). Similar data were obtained for SAS cells (Figure 3B). These data suggest that HMGA2 is related to the control of angiogenesis-related genes in OSCC cells.

### 2.6. Expression of HMGA2 and Angiogenesis-Associated Genes in Primary OSCC and Metastatic Tissue

**Figure 4:**
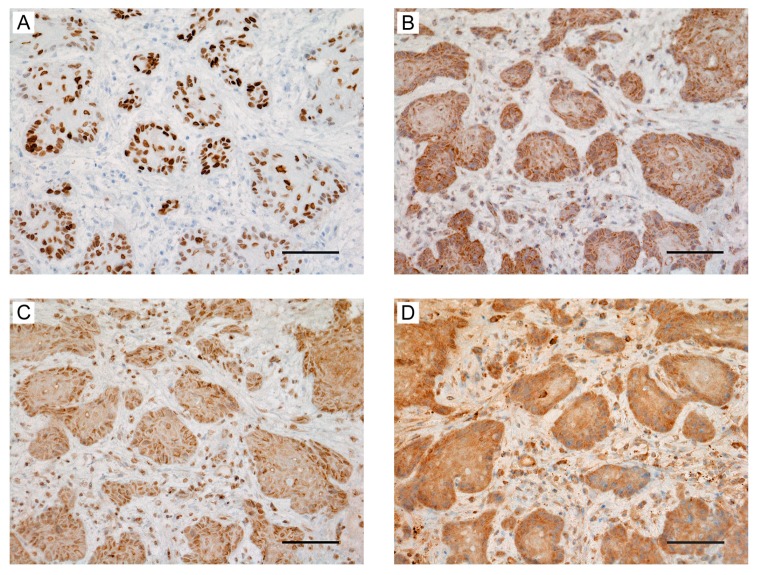
High expression of angiogenesis-associated genes in oral squamous cell carcinoma (OSCC) tissues based on HMGA2 expression. Images of immunohistochemical staining based on the following antibodies in OSCC biopsy specimens: (**A**) HMGA2, (**B**) VEGF-A, (**C**) VEGF-C, and (**D**) FGF-2. Scale bar, 100 μm.

**Figure 5:**
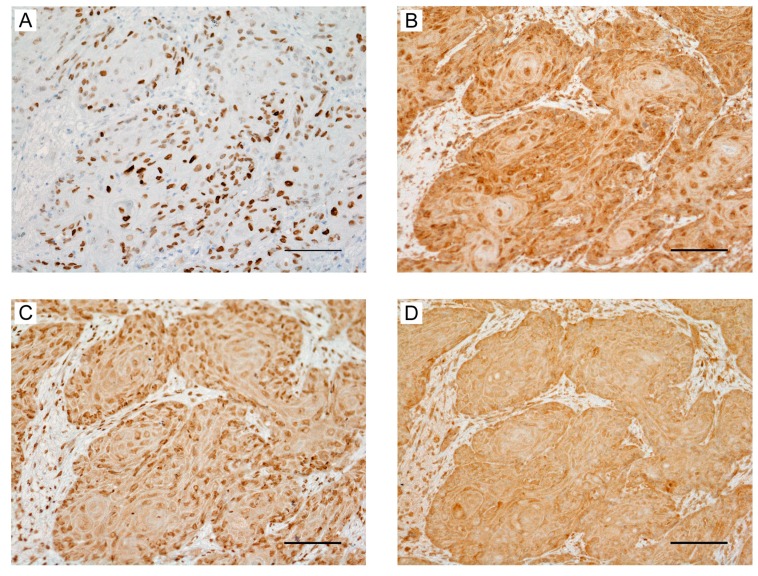
HMGA2 and angiogenesis-related genes are highly co-expressed at sites of lung metastasis. Images of immunohistochemical staining using the following antibodies in lung metastasis specimens: (**A**) HMGA2, (**B**) VEGF-A, (**C**) VEGF-C, and (**D**) FGF-2. Representative microscopic images are shown according to the expression status. Scale bar, 100 μm.

**Figure 6:**
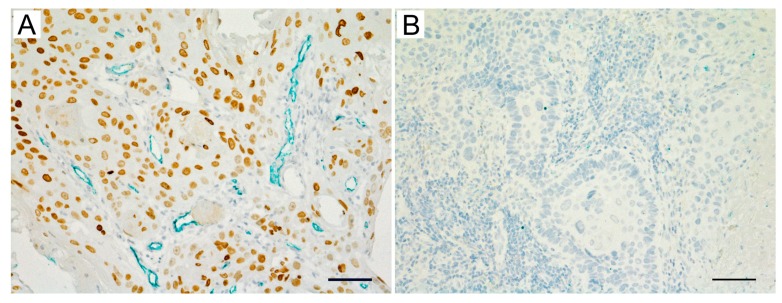
Blood vessels are increased in oral squamous cell carcinoma (OSCC) tissues with high HMGA2 expression. The association between blood vessel status, based on C34 staining and HMGA2 expression, as measured by immunohistochemical double staining with OSCC tissues. (**A**) HMGA2-high expression, (**B**) HMGA2-negative expression. CD34: green, HMGA2: brown. Scale bar, 100 μm.

To examine the association between HMGA2 and angiogenesis in OSCC tissues, the expression of associated molecules was analyzed through immunohistochemical staining. Tissue specimens comprised biopsy samples of primary tumors and lung metastasis obtained from OSCC patients. The expression of VEGF-A, VEGF-C, and FGF-2 was increased in primary samples of the high-HMGA2 expression group (Figure 4A–D). In contrast, the expression of VEGF-A, VEGF-C, and FGF-2 was decreased in primary samples of the low-HMGA2 expression group (Supplementary Figure S6A–D). Moreover, HMGA2, VEGF-A, VEGF-C, and FGF-2 were also highly expressed in lung metastasis samples (Figure 5A–D). Finally, we assessed the association between HMGA2 expression and the status of blood vessels in OSCC tissues. Blood vessels stained with CD34 were increased in the high HMGA2 expression group of OSCC tissues (Figure 6A,B). Taken together, these data suggest that HMGA2 is associated with distant metastasis through the regulation of angiogenesis-related genes in OSCC.

## 3. Discussion

The present study demonstrated the role of HMGA2 in clinical outcomes and the regulation of angiogenesis-associated genes in OSCC. As a new aspect regarding the function of HMGA2, we found that this protein is involved in the transcriptional regulation of angiogenesis-related genes in OSCC cell lines. Furthermore, we showed that high HMGA2 expression is associated with lymph node metastasis, distant metastasis, and poor prognosis in OSCC patients. To our knowledge, no other reports have identified the contribution of HMGA2 to both angiogenesis and distant metastasis in OSCC.

Previous reports indicated that high HMGA2 expression is involved in distant metastasis and poor prognosis in colorectal cancer [33,34]. Although, some reports described the association between HMGA2 overexpression and poor prognosis in oral cancer [27,28,29,30,31,32], no other reports have identified that high HMGA2 expression contributes to distant metastasis in OSCC. In this study, patients with high HMGA2 expression showed poor prognosis based on univariate analysis, although multivariate analysis demonstrated that HMGA2 expression was not an independent prognostic factor. It is considered that this result is due to the strong influence of distant metastasis as a prognostic factor. However, as mentioned, it was also implied that HMGA2 is a significant prognostic factor because HMGA2 expression and distant metastasis were found to be strongly correlated.

Cancer metastasis is regulated by a wide variety of molecules that contribute to certain events such as EMT and angiogenesis [4,35]. First, we assessed the relationship between HMGA2 and EMT in OSCC cells. It has been shown that HMGA2 is induced by the TGF-β pathway and can regulate transcriptional factors that control EMT, such as the zinc-finger proteins Snail and Slug and the basic helix-loop-helix protein Twist [8,22,36]. Consistent with these reports, our results showed that HMGA2 is up-regulated by TGF-β in OSCC cell lines. The high expression of HMGA2 mediated by TGF-β was found to facilitate EMT though the repression of E-cadherin and the activation of Vimentin. TGF-β treatment also markedly increased Slug but not Snail. In contrast, the depletion of HMGA2 increased E-cadherin and decreased Vimentin. Moreover, we found that Slug expression was positively regulated by HMGA2 in OSCC cell lines. Similar to our data, a previous report indicated that Slug expression is critical for the HMGA2-induced promotion of EMT in colon cancer [23]. However, the reduction of HMGA2 was not found to be associated with the repression of cell migration and invasion in OSCC cells. These data suggest that HMGA2 is partially involved in the EMT phenotype in OSCC cells. Second, we focused on the correlation between HMGA2 and angiogenesis, which is implicated in the development of distant metastasis. Angiogenesis, the process of forming new blood vessels, is a hallmark of tumor progression [15,37]. The ability of solid tumors including OSCC to stimulate angiogenesis is a critical feature that leads to tumor metastasis [14,38]. Although the VEGF family comprises well-known angiogenesis-inducers, the FGF family is also an important regulator of tumor angiogenesis [39]. Previous reports indicated that HMGA2 silencing suppresses the angiogenic behavior of endothelial cells and endothelial progenitor cells [25,26] and that HMGA proteins regulate FGF-2 in uterine fibroids [40]. Moreover, increased FGF-2 expression was correlated with the presence of metastasis and poor prognosis in oral tongue cancer [41]. However, little has been reported regarding the relationship between HMGA2 expression and angiogenesis in malignant tumors. For the first time, we report herein that the depletion of HMGA2 decreases the expression of angiogenesis-related markers such as VEGF-A, VEGF-C, and FGF-2 in OSCC cells. Moreover, in primary OSCC samples with high HMGA2 expression, angiogenesis genes were up-regulated and a high number of blood vessels was observed. These results indicate that HMGA2 might control tumor angiogenesis by regulating angiogenesis-associated genes in OSCC. 

However, an inherent limitation of our study is that the novel findings obtained were based on in vitro data, except for the immunohistochemical analysis using OSCC tissues. Therefore, further studies are needed to conform whether HMGA2 controls angiogenesis using in vivo models. We also need to clarify the molecular mechanisms underlying the pro-angiogenic function of HMGA2 in OSCC cells. This protein has three AT-hook DNA-binding motifs and can modulate transcription by changing the chromatin architecture. A previous report indicated that HMGA2 can epigenetically modulate the chromatin landscape of the *Cdh1* promoter during EMT [42]. Therefore, our aim is to demonstrate the epigenetic regulation of angiogenesis-related genes via HMGA2. Thus, further studies might suggest novel strategies to inhibit metastasis in OSCC, such as targeted molecular and epigenetic therapeutics.

In conclusion, we have highlighted the role of HMGA2 in clinical outcomes and the regulation of angiogenesis-associated genes in OSCC. Our data indicate that high HMGA2 expression could also represent a novel prognostic indicator. Moreover, therapies targeting HMGA2-dependent angiogenesis could represent a promising new approach to overcome metastasis in OSCC.

## 4. Materials and Methods 

### 4.1. Patients and Tissue Specimens

For clinicopathological analysis, primary oral cancer tissue samples were obtained from 110 patients with advanced OSCC treated at Kumamoto University Hospital between October 2003 and March 2013. All tumors were staged according to the TNM classification of the AJCC eighth edition (2017), and the degree of differentiation was determined according to the grade classification of the WHO. Tissue samples derived from biopsy specimens were used for immunohistochemical analyses. Lung tissues were obtained from OSCC patients confirmed to have lung metastasis. The samples were fixed with 10% formalin and embedded in paraffin. The study followed the guidelines of the Ethics Committee of Kumamoto University (the project identification code: RINRI No.1427, date of approval: 28 July 2018). The nature and aims of the study were explained to all patients, and all provided informed consent for the research.

### 4.2. Immunohistochemical Staining and Evaluation

Formalin-fixed paraffin-embedded specimens were cut into 4-μm sections and mounted on MAS-GP-coated slides (Matunami Glass Ind., LTD., Osaka, Japan). For HMGA2, the sections were heated in an autoclave with 0.01 mol/L citrate buffer (pH 7.0) for 15 min at 121 °C for antigen retrieval after deparaffinization and rehydration. The sections were incubated with 0.3% H_2_O_2_ in absolute methanol for 30 min to block endogenous peroxidase activity. Then, the sections were incubated with Protein Block Serum Free Reagent (Dako, Glostrup, Denmark) for 15 min to block nonspecific staining. After the blocking step was completed, the sections were incubated with antibodies against HMGA2 (D1A7; Cell Signaling Technology, Danvers, MA, USA), VEGF-A (RB-9031; Thermo Fisher Scientific, Waltham, MA, USA), VEGF-C (H-190, Santa Cruz Biotechnology, Inc., Dallas, TX, USA), FGF-2 (147, Santa Cruz Biotechnology, Inc., Dallas, TX, USA), and CD34 (BI-3C5, Santa Cruz Biotechnology, Inc., Dallas, TX, USA) at 4 °C overnight. This was followed by sequential 60-min incubations with the secondary antibodies (EnVision + System-HRP Labelled Polymer, Dako, Glostrup, Denmark) and visualization with the Liquid DAB+ Substrate Chromogen System (Dako, Glostrup, Denmark). All slides were lightly counterstained with hematoxylin for 30 s prior to dehydration and mounting.

### 4.3. Assessment of Immunohistochemical Staining

Three independent observers interpreted the immunohistochemical data in a blinded fashion. For each specimen, one score was assigned according to the percentage of positive cells as follows: <25%: 1 point; 25–50%: 2 points; 50–75%: 3 points; >75%: 4 points. Another score was assigned according to the intensity of staining, with negative staining set as 1, weak staining as 2, moderate staining as 3, and strong staining as 4. If the total expression score was ≥ 6, the lesion was considered to have high expression. 

### 4.4. Cell Lines

All human OSCC cell lines, namely Ca9-22 and SAS, were kindly donated by the Department of Oral and Maxillofacial Surgery, Graduate School of Dental Science, Kyushu University (Maidashi, Higashi-ku, Fukuoka, Japan). All cell lines were cultured in DMEM (Gibco, Grand Island, NY, USA) with 10% FBS. The cells were incubated at 37 °C with 5% CO_2_ and saturated humidity. 

### 4.5. Transfection with Small Interfering RNA and Reagent

Twenty-four hours before transfection, Ca9-22 and SAS cells were diluted in fresh medium without antibiotics and transferred to six-well plates. Cells were grown and transfected with scrambled siRNA (30 nM; Invitrogen, Carlsbad, CA, USA) using Lipofectamine RNAi MAX (Invitrogen, Carlsbad, CA, USA) as described in the manufacturer’s instructions. The cells were harvested 48 h post-transfection. 

### 4.6. Cell Proliferation Analysis

Cell proliferation was determined using the cell proliferation reagent WST-8 (Cell Counting Kit-8; Dojindo, Kumamoto, Japan). Briefly, 3 × 10^3^ cells were seeded in 96-well plates in 100 μL of medium in quadruplicate for each condition. At 24, 48, and 72 h, WST-8 reagent was added to each well at a 1:10 dilution and the plates were incubated for an additional 1 h at 37 °C. Sample absorbance was measured at 690/480 nm. Each experiment was performed in triplicate.

### 4.7. Scratch Wound Healing Assay

Cells were grown to reach 70–80% confluency in six-well plates. A linear wound was then generated by scraping the middle of the cell monolayer with a sterilized pipette tip. Cell migration to cover the wound space was examined and photographed at indicated time points (0 and 24 h) using a light microscope at 4× (Nikon, Japan). The migrated distance was analyzed by measuring the wound space at the aforementioned time points, and the wound width at time 0 was subtracted from these values. The migration values were indicated as a percentage of wound closure, and the control width at 0 h was set to 0%. Each independent experiment was repeated three times. 

### 4.8. Matrigel Cell Invasion Assay

Cell invasion activity was measured with the BioCoat Matrigel Invasion Chamber (Becton Dickinson, Tokyo, Japan) according to the manufacturer’s protocol. OSCC cells were inoculated on coated Matrigel in culture inserts (upper chamber) at a density of 2 × 10^5^ cells per 500 mL of serum-free DMEM. TGF-β1 (10 ng/mL; R&D Systems, Abingdon, UK) was then added to the culture insert (upper chamber) and incubated for 24 h at 37 °C and a 5% CO_2_ atmosphere. At the end of the incubation, the cells on the upper surface of the filter were completely removed with cotton swabs. The invaded cells that remained on the lower surface of the filter were fixed with methanol and stained with Diff-Quick (Sysmex, Hyogo, Japan). The numbers of stained cells in five randomly selected microscopic fields (×200) per filter were counted. The experiments were repeated at least three separate times. 

### 4.9. RNA Isolation, Reverse Transcription, and Quantitative PCR

Total RNA was isolated from cells using the RNeasy mini kit (QIAGEN AB, Sollentuna, Sweden) and cDNA synthesis was performed using the ReverTra Ace qPCR RT Master Mix (Toyobo, Osaka, Japan). 

For qualitative PCR, the cDNA was amplified using PCR with specific primers. The following primers were used: 

HMGA2forward: 5′-ACCCAGGGGAAGACCCAAA-3′ reverse: 5′-CCTCTTGGCCGTTTTTCT-3′E-cadherinforward: 5′-ATTTTTCCCTCGACACCCGAT-3′ reverse: 5′-TCCCAGGCGTAGACCAAGA-3′Vimentinforward: 5′-AGTCCACTGAGTACCGGAGAC-3′ reverse: 5′-CATTTCACGCATCTGGCGTTC-3′Slugforward: 5′-AAGCATTTCAACGCCTCCAAA-3′ reverse: 5′-GGATCTCTGGTTGTGGTATGACA-3′FGF-2forward: 5′-CACCTATAATTGGTCAAAGTGG-3′ reverse: 5′-CAGAAATTCAGTAGATGTTTCCC-3′VEGF-Aforward: 5′-CCTCCGAAACCATGAACTTT-3′ reverse: 5′-CCACTTCGTGATGATTCTGC-3′VEGF-Cforward: 5′-GCCCCAAACCAGTAACAATC-3′ reverse: 5′-GCTGGCAGGGAACGTCTAAT-3′GAPDHforward: 5′-CTGGGCTACACTGAGCACC-3′ reverse: 5′-AAGTGGTCGTTGAGGGCAATG-3′

For qRT-PCR, each reaction mixture was diluted five-fold with DNase/RNase-free water (Invitrogen, Carlsbad, CA, USA), and 4 μL of each mixture was subjected to PCR. The reactions were run using THUNDERBIRD SYBR qPCR Mix (Toyobo, Osaka, Japan) on a Light Cycler 1.5 (Roche, Indianapolis, IN, USA). The comparative *C*t (ΔΔ*C*t) method was used to determine fold-changes in expression using glyceraldehyde-3-phosphate dehydrogenase (GAPDH) as the reference. Each sample was run in triplicate. The cycling conditions were as follows: initial denaturation at 98 °C for 5 min, followed by 45 cycles at 98 °C for 15 s, 60 °C for 30 s and 72 °C for 60 s. The experiments were performed in triplicate.

### 4.10. Statistical Analysis

The differences in the mean values between the two groups were statistically analyzed using the Student’s t-test. For the analysis of HMGA2 levels in the tissue specimens, the chi-square test was used to determine the associations between the HMGA2 expression status and clinical and pathological variables. Survival analysis was performed according to the Kaplan–Meier method. The log-rank test was used to determine the correlations between HMGA2 expression status and patient survival. A multivariate survival analysis was performed using the Cox regression model to study the effects of HMGA2 expression on disease-free and overall survival. All p-values were based on two-tailed statistical analyses and p-values < 0.05 were considered statistically significant (* *p* < 0.05 and ** *p* < 0.01). All statistical analyses were completed using the JMP 9 software program (SAS Institute Inc., Cary, NC, USA). 

## Figures and Tables

**Table 1:** Correlation between HMGA2 expression and clinicopathological factors in 110 oral squamous cell carcinoma (OSCC) patients.

Characteristics	Total	HMGA2 Status		*p*-Value
		High Expression	Low Expression	
		*n* (%)	*n* (%)	
	110	42 (38.2)	68 (61.8)	
Age, years				
Median	67	65.9	67.7	
Range	30–87	30–85	33–87	
≤65	45	19 (42.2)	26 (57.8)	0.468
>65	65	23 (35.4)	42 (64.6)	
Sex				
Male	66	28 (42.4)	38 (57.6)	0.262
Female	44	14 (31.2)	30 (68.2)	
Primary site				
Tongue	34	13 (38.2)	21 (61.8)	0.532
Mandible gingiva	25	12 (48.0)	13 (52.0)	
Maxilla gingiva	21	8 (38.1)	13 (61.9)	
Buccal mucosa	16	4 (25.0)	12 (75.0)	
Oral floor	13	4 (30.8)	9 (69.2)	
Palate	1	1 (100)	0 (0)	
T-stage				
T1, T2	37	11 (29.7)	26 (70.3)	0.08
T3	25	7 (28.0)	18 (72.0)	
T4	48	24 (50.0)	24 (50.0)	
N-stage				
N0	34	7 (20.6)	27 (79.4)	0.011 *
≥N1	76	35 (46.1)	41 (53.9)	
Clinical stage				
II	13	2 (15.4)	11 (84.6)	0.142
III	26	9 (34.6)	17 (65.4)	
IV	71	31 (43.7)	40 (56.3)	
Differentiation				
Well	90	35 (38.9)	55 (61.1)	0.746
Moderate	20	7 (35.0)	13 (65.0)	
Local recurrence				
Yes	28	11 (39.3)	17 (60.7)	0.889
No	82	31 (37.8)	51 (62.2)	
Distant metastasis				
Yes	16	13 (81.3)	3 (18.7)	<0.001 **
No	94	29 (30.9)	65 (69.1)	

The chi-square test was used to examine the correlation between HMGA2 expression and clinicopathological factors in 110 OSCC patients. ** *p* < 0.01, * *p* < 0.05.

**Table 2:** Results of the multivariate analysis of prognostic factors by the Cox proportional hazards regression model comparing oral squamous cell carcinoma patients based on HMGA2 expression.

Characteristics	Assigned Score	OS		DFS	
		Hazard Ratio (95% CI)	*p*-Value	Hazard Ratio (95% CI)	*p*-Value
HMGA2 expression					
Low	0	1.254 (0.528–3.012)	0.609	1.195 (0.545–2.650)	0.657
High	1				
N-stage					
N0	0	1.290 (0.508–3.692)	0.603	1.068 (0.455–2.639)	0.882
≥N1	1				
Local recurrence					
No	0	10.33 (4.640–24.72)	<0.001 **	14.09 (6.553–32.68)	<0.001 **
Yes	1				
Distant metastasis					
No	0	27.89 (10.09–80.16)	<0.001 **	5.222 (2.247–12.19)	<0.001 **
Yes	1				

Abbreviations: CI = confidence interval. * *p* < 0.05, ** *p* < 0.01.

## Supplementary Materials

Supplementary materials can be found at https://www.mdpi.com/1422-0067/20/10/2473/s1.

## Abbreviations

EMT	Epithelial–mesenchymal transition
HMGA2	High-mobility group A protein 2
OSCC	Oral squamous cell carcinoma
qRT-PCR	quantitative RT-PCR
