# Supplementary material for: HMGA2 Contributes to Distant Metastasis and Poor Prognosis by Promoting Angiogenesis in Oral Squamous Cell Carcinoma
Source: Int J Mol Sci. 2019 May 19;20(10):2473. doi: 10.3390/ijms20102473 (PMC6566167; doi:10.3390/ijms20102473)
Supplement: Supplementary file 1 [file ijms-20-02473-s001.pdf]

Supplementary Materials

HMGA2 Contributes to Distant Metastasis and Poor Prognosis by Promoting Angiogenesis in Oral Squamous Cell Carcinoma

Junki Sakata [1,†], Akiyuki Hirosue [1,*,†], Ryoji Yoshida [1,†], Kenta Kawahara [1], Yuichiro Matsuoka [1], Tatsuro Yamamoto [1], Masafumi Nakamoto [1], Masatoshi Hirayama [1], Nozomu Takahashi [1], Takuya Nakamura [1], Hidetaka Arita [1], Hikaru Nakashima [1], Masashi Nagata [1], Akimitsu Hiraki [2], Masanori Shinohara [1] and Hideki Nakayama [1]

[1] Department of Oral and Maxillofacial Surgery, Faculty of Life Sciences, Kumamoto University, Kumamoto 860-8556, Japan; j.sakata0510@gmail.com (J.S.); ryoshida@kumamoto-u.ac.jp (R.Y.); k.k.ronbun@gmail.com (K.K.); kokopelli205@gmail.com (Y.M.); tatsuro.y.1.3@gmail.com (T.Y.); mabo.20jd.jm@gmail.com (M.N.); dd104040@gmail.com (M.H.); n.takahashi6074@gmail.com (N.T.); takuyanakamura2008@gmail.com (T.N.); hidetaka.arita@gmail.com (H.A.); ricken_hikaru@yahoo.co.jp (H.N.); nagatama0213@gmail.com (M.N.); shinora@kumamoto-u.ac.jp (M.S.); hinakaya@kumamoto-u.ac.jp (H.N.)

[2] Section of Oral Oncology, Department of Oral and Maxillofacial Surgery, Fukuoka Dental College, Fukuoka 814-0193, Japan; hiluckyhilucky@gmail.com

* Correspondence: ahiro711@kumamoto-u.ac.jp; Tel.: +81-96-373-5288

† These authors contributed equally to this work.

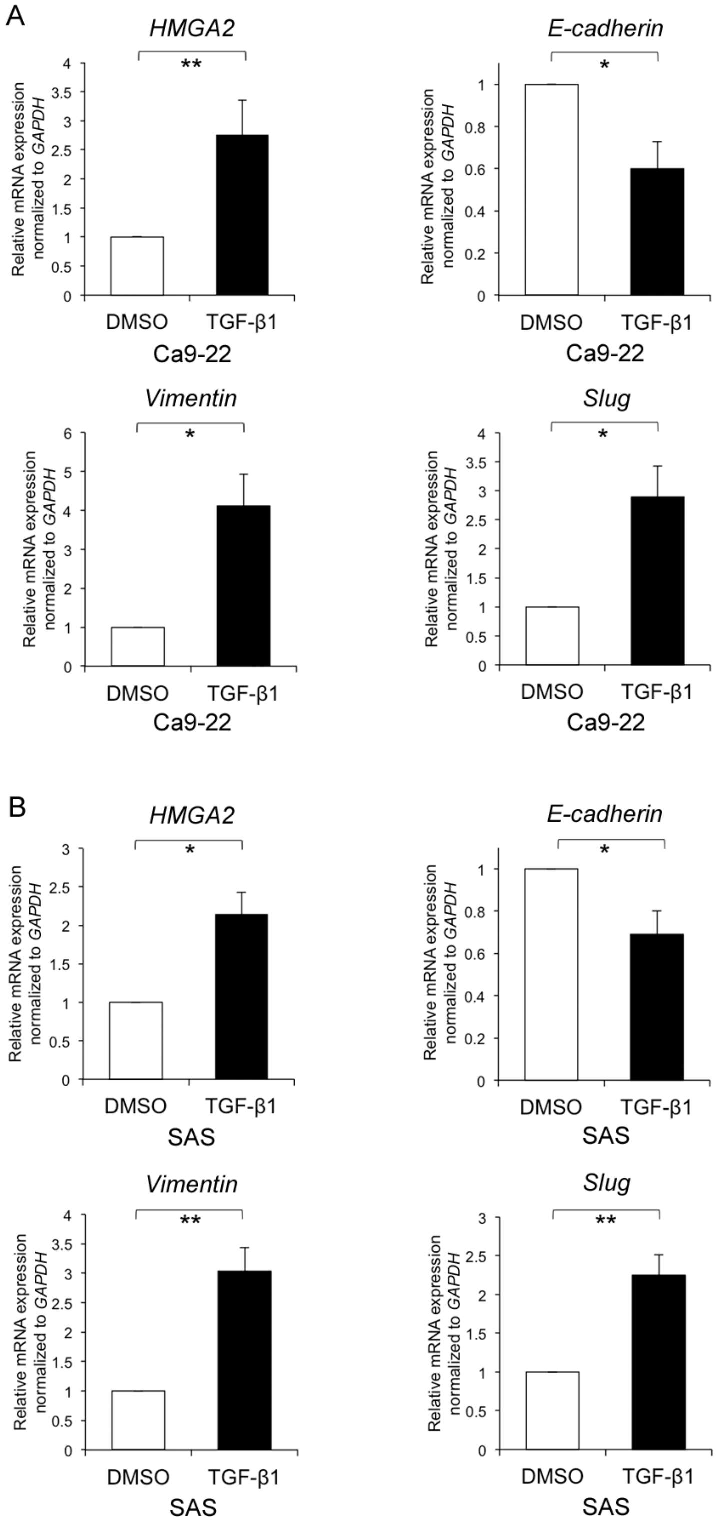

Supplementary Figure S1. HMGA2 and epithelial–mesenchymal transition (EMT) markers are overexpressed upon TGF-β1 stimulation. The mRNA expression levels of *HMGA2*, *Slug*, *E-cadherin*, and *Vimentin* in (A) Ca9-22 and (B) SAS cells stimulated with TGF-β1 for 48 h. *p < 0.05, **p < 0.01.

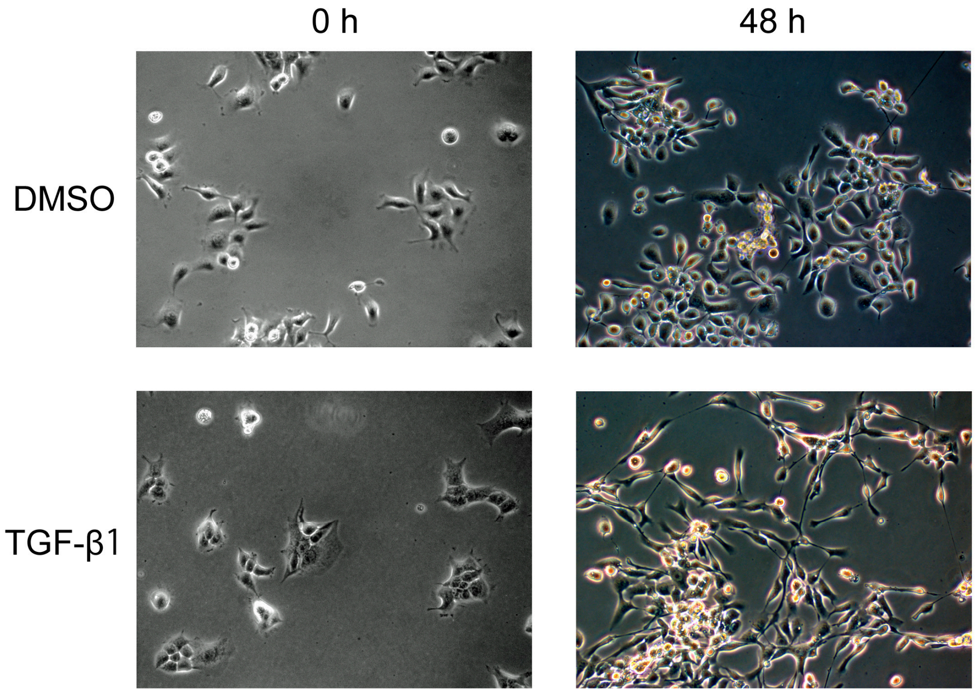

Supplementary Figure S2. TGF-β1 stimulation induces a morphological change from the cobblestone form to the spindle shape in oral squamous cell carcinoma (OSCC) cells. Phase-contrast images of Ca9-22 cells stimulated with DMSO (control) or TGF-β1 for 0 and 48 h. Scale bar, 100 μm.

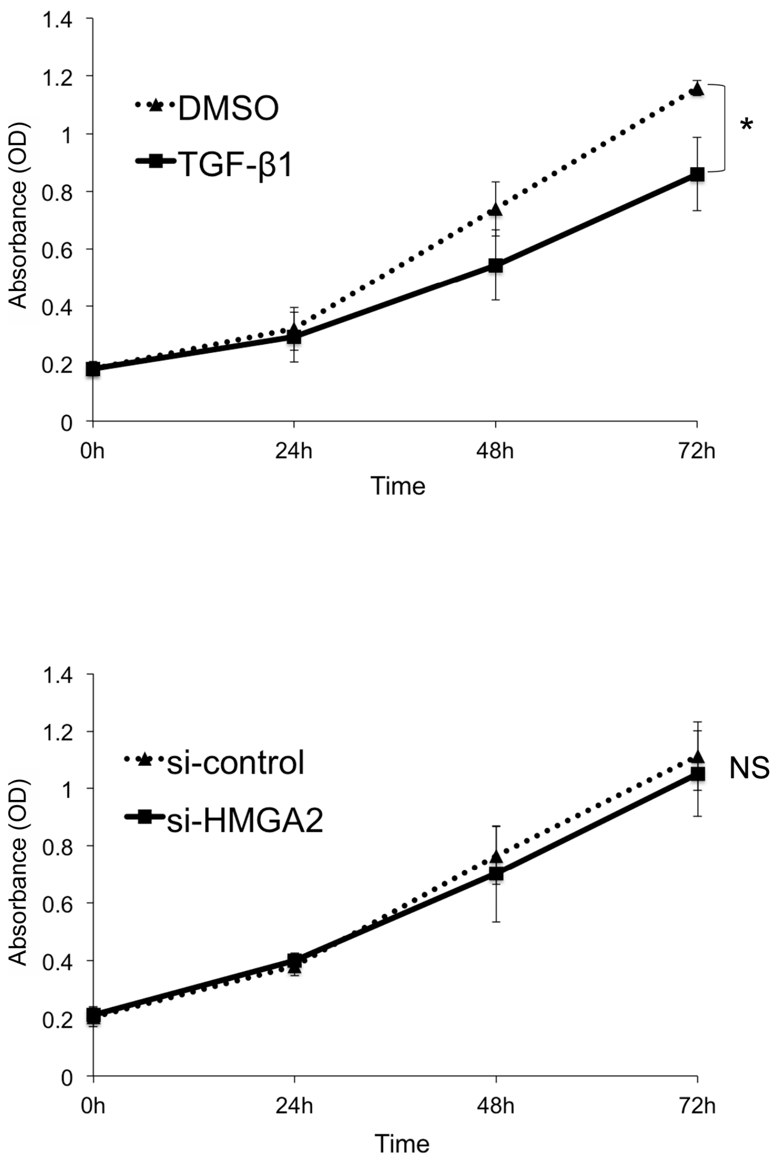

Supplementary Figure S3. Cell proliferation is inhibited by TGF-β1 stimulation but not HMGA2 suppression. Cell proliferation assay with Ca9-22 cells (A) stimulated with DMSO (control) and TGF-β1, and (B) transfected with si-control and si-HMGA2. Statistical analysis was performed using a Student's t-test. *$p < 0.05$.

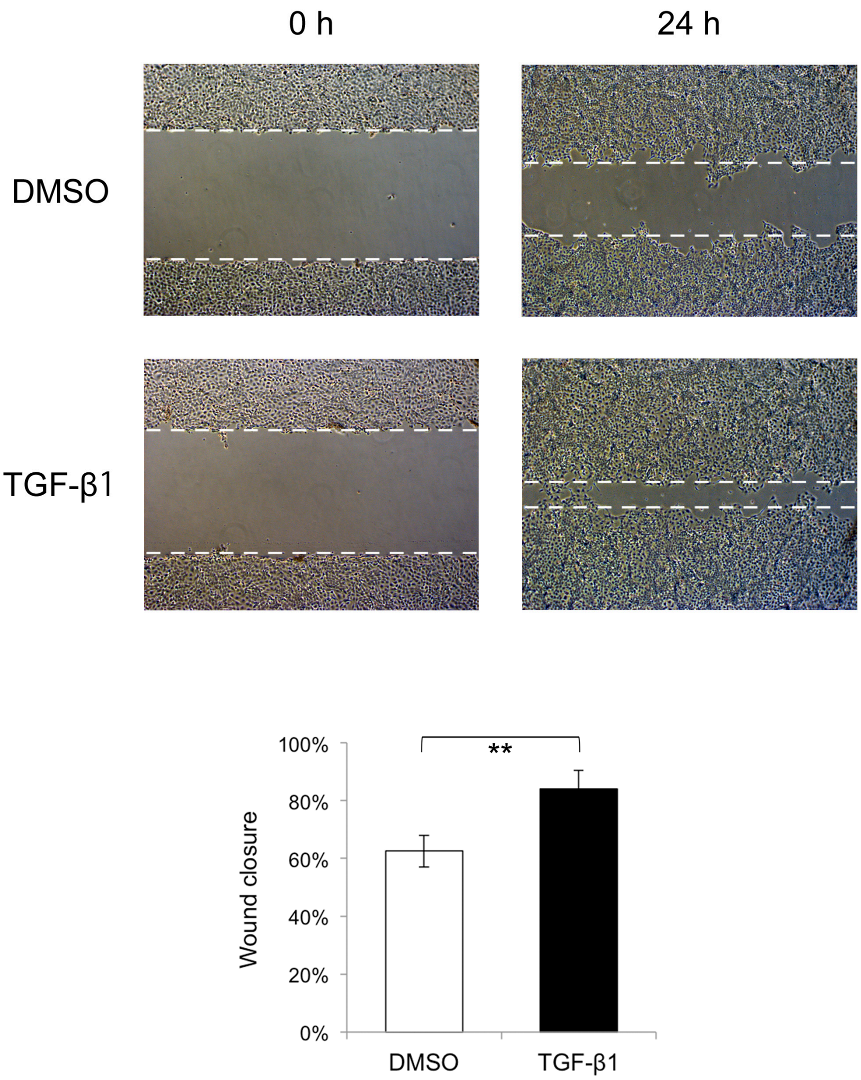

Supplementary Figure S4. Cell migration is promoted by TGF-β1 stimulation. Wound healing assay with Ca9-22 cells stimulated with DMSO (control) and TGF-β1. The wound space was photographed at 0 and 24 h. The vertical distance of the wound was evaluated and statistically analyzed. Statistical analysis was performed using a Student's t-test. **$p < 0.01$.

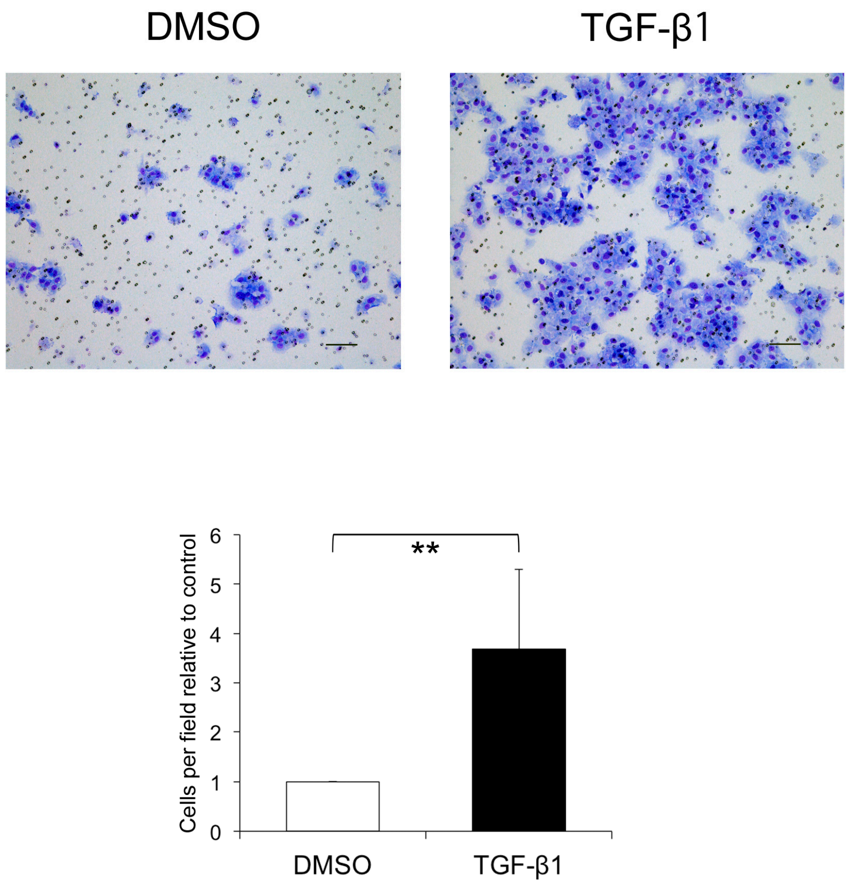

Supplementary Figure S5. Cell invasion is promoted by TGF-β1 stimulation. Cell invasion assay with Ca9-22 cells stimulated with DMSO (control) and TGF-β1. The images are representative fields of invasive cells on the membranes. Statistical analysis was performed using a Student's t-test. $^{**}p < 0.01$.

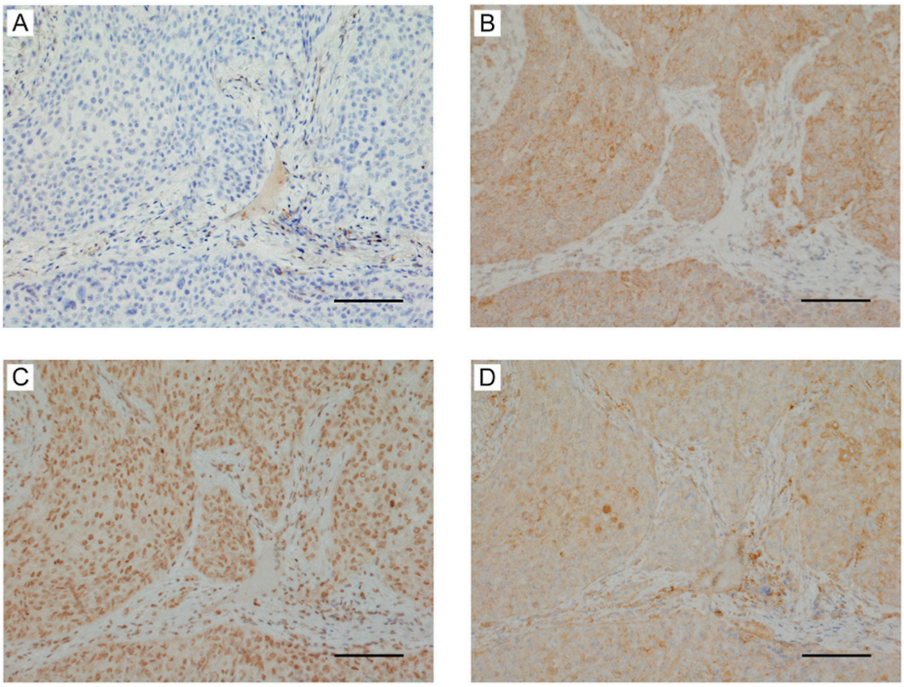

Supplementary Figure S6. The expression of angiogenesis-associated genes is decreased in oral squamous cell carcinoma (OSCC) tissues expressing low levels of HMGA2. Immunohistochemical staining using the follow antibodies on OSCC biopsy specimens: (A) HMGA2, (B) VEGF-A, (C) VEGF-C, and (D) FGF-2. Scale bar, 100 μm.
